# Investigation of the Serotonergic Activity and the Serotonin Content in Serum and Platelet, and the Possible Role of the Serotonin Transporter in Patients with Depression

**DOI:** 10.3390/bs12060178

**Published:** 2022-06-03

**Authors:** Jasmin Obermanns, Vera Flasbeck, Saskia Steinmann, Georg Juckel, Barbara Emons

**Affiliations:** 1 LWL University Hospital, Department of Psychiatry, Psychotherapy and Preventive Medicine, Ruhr University Bochum, Alexandrinenstraße 1-3, 44791 Bochum, Germany; jasmin.obermanns@rub.de (J.O.); georg.juckel@rub.de (G.J.); 2 LWL University Hospital, Department of Psychiatry, Psychotherapy and Preventive Medicine, Division of Social Neuropsychiatry and Evolutionary Medicine, Ruhr University Bochum, Alexandrinenstraße 1-3, 44791 Bochum, Germany; vera.flasbeck@rub.de; 3 Psychiatry Neuroimaging Branch, Department of Psychiatry and Psychotherapy, University Medical Center Hamburg-Eppendorf, Martinstraße 52, 20246 Hamburg, Germany; s.steinmann@uke.de

**Keywords:** depression, serotonin, platelet, serotonergic activity, 5-HTTLPR

## Abstract

According to the monoamine hypothesis, the development of depression is associated with dysfunctions of the serotonergic system. Alterations in the serotonin transporter gene (5-HTTLPR), the serotonergic activity in the brain, and the content of serotonin (5-HT) have been related to depression and were examined separately by previous studies. This study investigates these parameters in 89 depressed patients and 89 healthy participants. We investigated the serotonergic activity measured by the loudness dependence of auditory evoked potentials (LDAEP). In addition to the examination of the serotonin content (serum and platelet), enzyme-linked immunosorbent assays (ELISA) were used and 5-HTTLPR genotypes were analyzed. We observed a lower serotonin content in patients compared to healthy participants. Further, we noticed a correlation between anxiety and depression-associated symptoms with serotonergic activity. Patients treated with SSRI/SNRI showed decreased contents of serum serotonin compared to patients without any psychotropic medication or other psychotropic medications. Since the serotonergic activity, peripheral serotonin content, and 5-HTTLPR were unrelated, the results suggest independent alterations of central and peripheral serotonergic systems in depression. In line with this finding, serotonergic activity was related to anxiety and depression symptoms. Furthermore, the applied medication seems to influence serum serotonin content in patients with depression.

## 1. Introduction

Major Depressive Disorder (MDD) is one of the most common and serious psychiatric diseases [1]. Currently, the monoamine hypothesis offers the most reliable explanation regarding the development of MDD [2,3], suggesting a dysregulation of the serotonergic system in the central nervous system (CNS). The etiology of depression cannot be explained only by the monoamine hypothesis; it consists of different factors and influences that have not been fully elucidated [4,5].

Besides the serotonergic system in the CNS, a platelet serotonin system has been discovered. Despite some similarities, these two systems differ concerning metabolizing enzymes. Platelets are not able to synthesize serotonin because of the lack of tryptophan hydroxylase [6,7,8]. Regarding psychopathology, previous studies have shown lower serotonin levels in platelets in patients suffering from depression [8,9,10].

Alterations in genes involved in the serotonergic system have also been considered an important player in the etiology of depression. One of the most studied genetic polymorphisms associated with depression is the 5-HTTLPR (5-HTT linked polymorphic region) [11,12], which is a functional length polymorphism in the promoter region of the serotonin transporter gene (SLC6A4) [13]. The 5-HTTLPR generates a short allele (s-allele) and a long allele (l-allele) [14,15]. S-allele reduces the expression of the SLC6A4 transcription and is associated with higher susceptibility to psychiatric disorders [16,17,18].

Besides serotonin levels and alterations in genes involved in serotonergic functioning, serotonergic neurotransmission could also be a crucial aspect of depression. Variations in serotonin neurotransmission may increase the risk of psychiatric disorders. The “loudness dependence of auditory evoked potentials” (LDAEP)—a non-invasive method may be applied to measure its activity. Preclinical and animal studies have shown that LDAEP is an indicator of serotonergic activity in the brain [19,20,21,22,23]. Additionally, Kawohl, and colleagues (2008) showed a negative correlation between LDAEP and the peripheral serotonin level in serum [24].

Overall, previous research proposed multifaceted alterations in the serotonergic system in patients suffering from depression, including peripheral and CNS serotonin levels as well as variations in genotype and serotonergic activity. Thus, many different approaches exist to investigate the development of depression. However, previous studies mainly investigated the abovementioned parameters in isolation and did not assess interactions.

The present study aimed to find correlations between the serotonin content, the serotonergic activity, and the 5-HTTLPR genotype in patients with depression. Lastly, we estimate a dysregulation of the serotonergic system in depressed patients.

## 2. Materials and Methods

### 2.1. Cohort and Study Design

For the current study, we included 89 patients (57 female and 32 male, average age 37 ± 12) with a depressive disorder and 89 matched healthy participants (57 female and 32 male, average age 37 ± 12). All individuals had given their written informed consent. According to the International Statistical Classification of Diseases and Related Health Problems (ICD-10), all patients included in the study had depression (F31.3/F31.4: bipolar affective disorder; F32.0/F32.1/F32.2: depressive episode; F33.0/F33.1/F33.2: recurrent depressive disorder). Patients with any comorbidity were excluded. All participants were of age and not older than 60 years old. Furthermore, healthy participants were only included in the study when they had no psychiatric disorders. Patients and healthy participants who abused drugs or alcohol, and exhibited neurological or serious internal diseases were excluded. The patients were recruited at the LWL University Hospital for Psychiatry, Psychosomatic and Preventive Medicine in Bochum, Germany. The study was accepted by the Ethics Committee of the Medical Faculty of the Ruhr University Bochum (ethic application number 5121-14) and in accordance with the Declaration of Helsinki (1975 revised in 2008). The patients were treated as inpatients or as outpatients. The treatments of the patients were carried out by psychotherapy and psychopharmacologic medications. The clinical, social, and demographic characteristics of the subjects are shown in Table 1. The applied medications and applied dosage are shown in Table 2. The detailed derivation of the applied medication is shown in Supplementary Materials Table S2.

In the current study, patients and healthy participants underwent a fasting blood withdrawal in the morning to measure the serotonin content in serum and platelet, and to determine the 5-HTTLPR genotype. Moreover, an EEG was recorded to measure the serotonergic activity. Additionally, the patients and the healthy participants completed psychometric questionnaires to exclude a psychiatric disease in the group of healthy participants and to confirm a depression in the group of patients.

### 2.2. Psychometric Instruments

All patients and healthy participants completed a psychometric questionnaire and participated in a neuropsychiatric interview (M.I.N.I) for classification purposes [25]. The severity of depression was assessed by the Hamilton Depression Scale (HAMD) [26]. The division of the severity of depression by HAMD is defined as follows: no depression (scores 0–9), slight depression (scores 10–19), moderate depression (scores 20–29), serious depression (scores ˃ 30) [26]. In addition, the Beck Depression Inventory (BDI) [27] and State–Trait Anxiety Inventory (STAI) [28] were used as self-rating questionnaires. The classification of the severity of depressive pathology by the BDI was defined as follows: no depression (scores 0–8), slight depression (scores 9–19), moderate depression (scores 20–28), serious depression (scores ˃ 29) [27].

### 2.3. Serotonin Measurements

Whole blood samples were collected from each patient and the healthy participant by venipuncture into ethylenediaminetetraacetic acid (EDTA) tubes and serum tubes (Sarstedt, Nümbrecht, Germany). Serum tubes were kept frozen at −80 °C until assayed.

To measure the platelet serotonin, the blood was immediately centrifuged at 200× *g* for 10 min (min) (at room temperature (rt)). The platelet-rich plasma (PRP) was transferred into a new tube (Sarstedt, Nümbrecht, Germany) and mixed carefully.

To count the number of cells in the “Neubauer Improved” chamber, 20 μL of the supernatants were mixed with 20 μL tryphan blue and filled into the “Neubauer Improved” chamber. Dead cells turned blue and living cells remained clear. The living cells were counted using the squares in the “Neubauer Improved” chamber and the cell number was calculated.

The PRP was centrifuged again (2250× *g*, 20 min, 4 °C) to obtain the platelet-poor plasma (PPP), which was disposed of. The cell pellet was resuspended with sodium chloride (3 mL) and centrifuged (2250× *g*, 20 min, 4 °C). The supernatant was discarded. The pellet was washed again with sodium chloride (3 mL, 9%) and centrifuged again (2250× *g*, 20 min, 4 °C). After the last centrifugation step, the supernatant was removed, and the pellet was resuspended in sodium chloride (1 mL, 9%). Subsequently, the pellet was adjusted to a cell count of 6.6 × 10^6^ cells/mL and carefully resuspended with Aqua bidest. The sample was aliquoted and kept frozen at −80 °C until further examination.

Serum and platelet serotonin content was measured by using the Serotonin ELISA kit from Enzo Life Science (Farmingdale, NY, USA). The content was measured according to the manufacturer’s instructions and calculated by using a standard straight line.

### 2.4. Genotyping of the 5-HTTLPR

The genomic DNA of patients and healthy participants was extracted from EDTA tubes (Sarstedt, Germany) using the Qiagen Blood Mini Kit (Qiagen, Hilden, Germany) according to the manufacturer’s instructions. The analysis of the 5-HTTLPR was performed by a qualitative PCR followed by gel electrophoresis, according to the method of Wendland et al. (2006) [29].

### 2.5. Loudness Dependence of Auditory Evoked Potentials (Cortical LDAEP)

**Figure 1:**
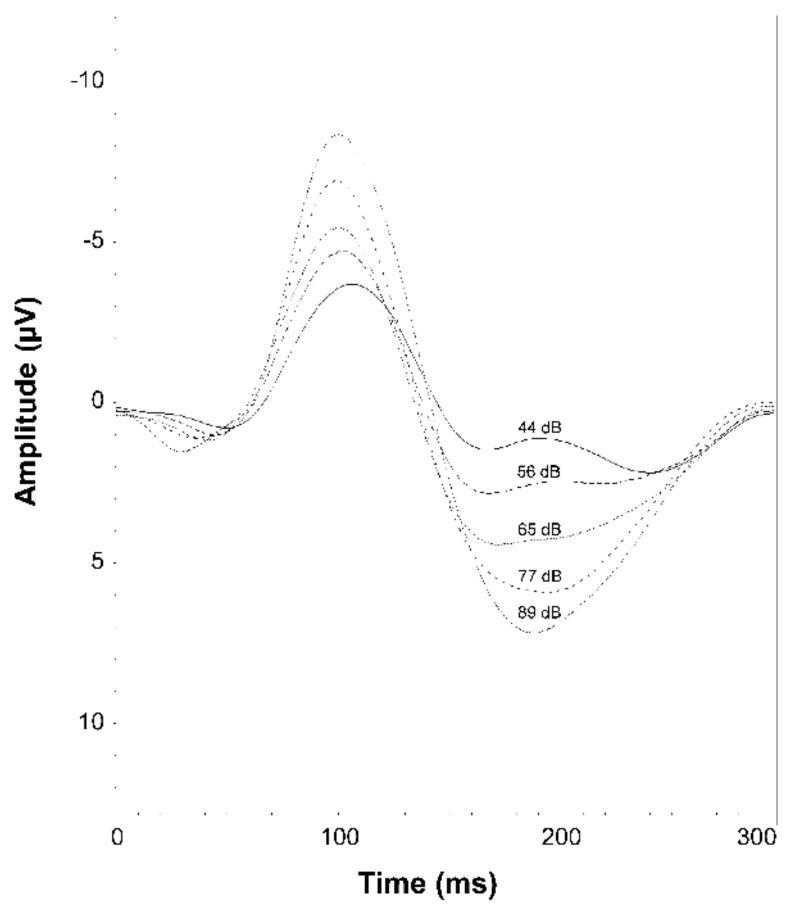
Grand mean auditory evoked potentials at CZ for the patients and healthy participants. The different intensities of the auditory stimuli at 44, 56, 65, 77, and 89 dB.

All subjects were seated in a chair in a sound-attenuated room with eyes open. Obtained interstimulus intervals were between 1800 and 2200 ms. Tones at 1000 Hz with a duration of 40 ms, including five loudness levels (44, 56, 65, 77, 89 dB sound pressure level) (Figure 1) were generated by Presentation^®^ software (Neurobehavioral Systems, Inc., Version 14.9; Berkeley, CA, USA) and presented via earphones (Sony Stereo Headphones MDR-1A, Sony^®^ Corporation, Tokyo, Japan).

For the EEG acquisition, 32 non-polarized silver-silver chloride electrodes were attached to a suitable EEG cap (Easy Cap^®^, Woerthsee-Etterschlag, Germany) according to the 10/20 system (impedance ≤ 10 kΩ). The EEG was recorded with a sampling rate of 250 Hz and an analog band-pass filter from 0.531–70 Hz using the Brain Vision BrainAmp^®^ MR (Brain Products GmbH, Munich, Germany). Data were analyzed using the BrainVision Analyzer 2.0 (Version 2.01.3931; Brain Products GmbH, Gilching, Germany) with a bandpass filter from 0.5–20 Hz. Epochs with excessive eye or body movements (±100 μV) were automatically rejected, and the residual sweeps were reduced. Only participants with at least 40 artifact-free trials per intensity were included in the further analysis. A semi-automatic measurement on the Cz electrode occurred at maximum values of N1 (50–150 ms) and P2 (100–250 ms). The N1/P2 amplitude was calculated as the difference of peak amplitude between N1 and P2. The calculation of the LDAEP was determined by the median of all slopes of each possible connection between the five different N1/P2 amplitudes [19].

### 2.6. LORETA (Source LDAEP)

Based on the scalp-recorded EEG, the standardized low-resolution brain electromagnetic tomography (sLORETA) was used to estimate the cortical current density [30]. This technique offers the possibility to calculate an electromagnetic brain tomography with a low resolution from the average EEG data. The sLORETA is based on the Montreal Neurological Institute average MRI brain map (MNI 152 template). The solution space is defined as the hippocampus and the cortical grey matter. The cortical volume is partitioned in 6239 voxels of 5 mm spatial resolution. For this sLORETA analysis, data were re-referenced to the average of all electrodes. The sLORETA was used to calculate the cortical current density of the LDAEPs of all five stimuli for every subject within a time window from 50 to 250 ms post-stimulus. Region-of-interest (ROI) analyses were performed for the right and left Heschl’s gyrus (BA41). The BA41-ROIs consisted of all voxels of the predefined regions from the LORETA software package [31]. The source LDAEP was calculated as the slope of the linear regression of the cortical current density.

### 2.7. Statistical Analysis

To determine the correlations between the two groups, Pearson’s correlation was used. The matching criteria of the patients and healthy participants were based on age and gender. For the correlation, we included only the matched pairs where all values were present (values of serotonin content in platelet n = 72/serum n = 88; values of cortical/source LDAEP n = 66). Reasons for non-included matched pairs contained missing values of the analyzed variables, resulting in the absence of blood withdrawal or technical reasons. To calculate the correlation between the serotonergic activity, the 5-HTTLPR, psychometric, and the peripheral serotonin content bivariate correlation were used. A partial correlation was used to estimate the influence of medication on the above-mentioned variables. The distribution and presence of the Hardy–Weinberg equilibrium was tested with the chi-squared test (χ^2^-test) for best fit. The χ^2^-test was used to determine the statistical significance of the difference between the allele frequencies. A difference between the groups was tested with an independent two-sample *t*-test. The influence of confounding variables (age, gender, severity of depression, nicotine use, medication, marital status) was calculated with an ANCOVA or a MANCOVA. Statistical analysis was performed using IBM^®^ SPSS^®^ Statistic Software (IBM Corp., Version 25.0. Armonk, NY, USA). The influence of confounding variables was proven by ANCOVA and MANCOVA. The data of all participants regarding the serotonin content, serotonergic activity, psychometric questionnaires, and 5-HTTLPR genotyping are shown in Supplementary Materials (Table S1).

## 3. Results

### 3.1. Psychometry

The comparison of the psychometric questionnaires showed that patients with depression reached higher scores in the Hamilton (HAMD-21), exhibiting slight (scores 10–19) to moderate depression scores (20–29). The Beck Depression Inventory (BDI-II) revealed a similar result, showing that patients were suffering from moderate (scores 20–28) to serious depression (scores ˃ 29). In both questionnaires, the healthy participants reached low scores indicating no depression. According to the State–Trait Anxiety Inventory (STAI-X1/STAI-X2), patients with depression showed significantly higher scores compared to the healthy participants (Table 3).

### 3.2. Comparison of Serotonin Levels in Platelet and Serum in Healthy Participants and Patients with Depression

**Figure 2:**
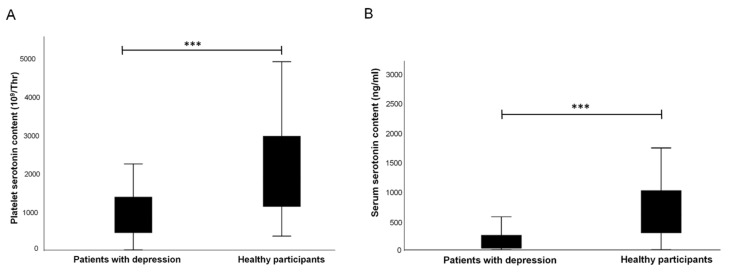
(**A**). Comparison of platelet serotonin content in patients and healthy participants. (**B**). Comparison of the serum serotonin content in patients and healthy participants. *** *p* ˂ 0.001.

The serotonin levels in platelet and serum were significantly reduced in patients compared to the healthy participants (platelet *t*(142) = −5.4, *p* = < 0.001; serum *t*(172) = −6.1, *p* = < 0.001; Table 3; Figure 2).

**Figure 3:**
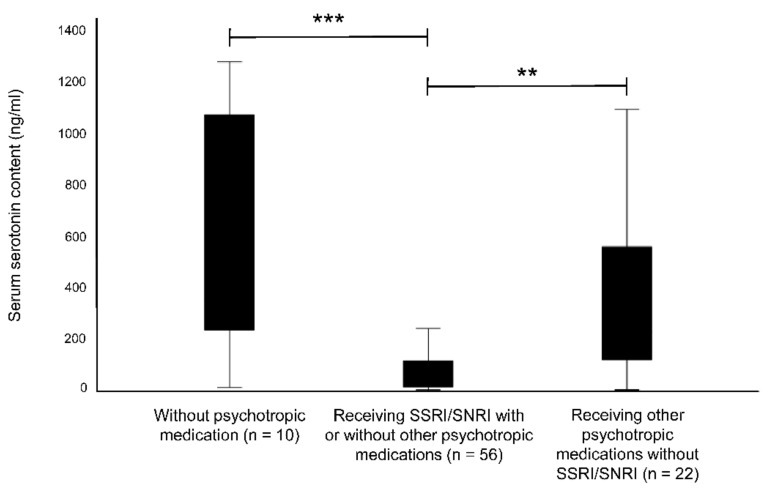
Comparison of serum serotonin content in patients without psychotropic medication and patients receiving SSRI/SNRI or other psychotropic medications without SSRI/SNRI. ** *p* ˂ 0.01; *** *p* ˂ 0.001.

Further analysis represented significant differences in serum serotonin content between patients who received selective serotonin reuptake inhibitors (SSRI) or selective norepinephrine reuptake inhibitors (SNRI) to those without any psychotropic medication (*t*(64) = 4.489, *p* ˂ 0.001) or received other psychotropic medication excluding SSRI/SNRI (*t*(76) = −3.154, *p* = 0.002) (Figure 3). The comparison of patients with no psychotropic medications and those treated with other psychotropic medications without SSRI/SNRI showed no significant variation. A difference between these groups was not significant in platelet serotonin content. Additional analyses regarding the different medical treatments, the serotonergic activity, and psychometry are shown in Supplementary Materials (Table S3).

**Figure 4:**
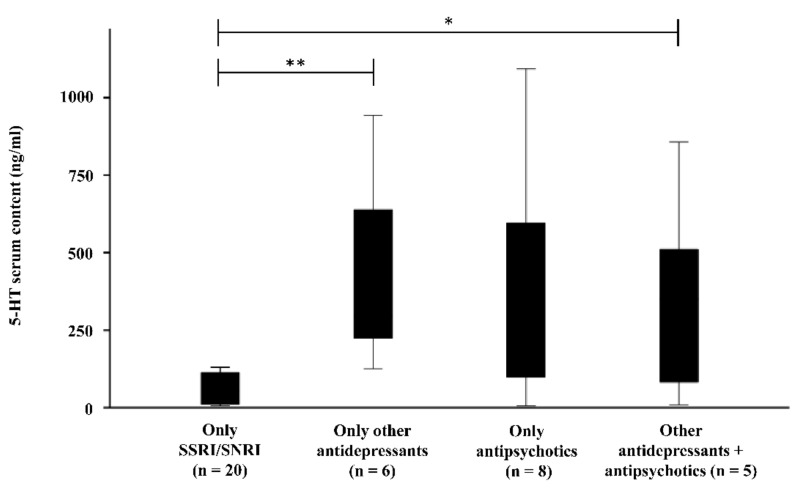
Comparison of 5-HT serum content in patients who received only SSRI/SNRI to patients treated only with other antidepressant (tri-/tetracyclic antidepressants, melatonin receptor agonists), only antipsychotics, or a combination of other antidepressants and antipsychotics. * *p* ˂ 0.05; ** *p* ˂ 0.01.

Reduced serum serotonin levels were found in patients treated only with SSRI/SNRI compared to patients treated with other antidepressants (tetra-/-tricyclic antidepressants, melatonin receptor agonist) (SSRI/SNRI (n = 20): 111.09 ± 187.18 ng/mL; other antidepressants (n = 6): 420.16 ± 311.03 ng/mL; *t*(24) = −3.034; *p*= 0.006)) or a combination of antipsychotics and other antidepressants (antipsychotics (n = 5): 362.24 ± 342.80 ng/mL; *t*(23) = −2.260; *p* = *0*.034) (Figure 4). The comparison of antipsychotics and SSRI/SNRIs showed no significant difference, probably due to the small sample size (n = 8; 362.14 ± 371.64 ng/mL; *t*(8.46) = −1.820; *p* = 0.104). A difference between medication and platelet serotonin content could not be detected in patients.

Regarding the serotonin content in platelets, a positive correlation with the serotonin content in the serum of the group of healthy participants (Table 4) was found. Interestingly, in the group of healthy participants, platelet serotonin levels correlated negatively with the HAMD-21 and the STAI-X2 (Table 4). When age and gender were included as confounding variables, no effect emerged on the peripheral serotonin content.

The significant results are summarized graphically in the Supplemental Material Figure S1.

### 3.3. Serotonergic Activity (Cortical and Source LDAEP)

The comparison of cortical LDAEP values between the patients with depression and healthy participants showed no significant difference (Table 3).

The results gained from source LDAEP, calculated by sLORETA, showed no significant differences between the patients and healthy participants (Table 3).

Interestingly, the analysis of the correlation between the self-questionnaires BDI-II and STAI-X1 with the cortical LDAEP revealed an association only in the group of patients (Table 4). In healthy participants, these correlations were not significant. A positive relation between the source LDAEP and cortical LDAEP (right hemisphere r = 0.488; *p ≤* 0.001; left hemisphere r = 0.633; *p* ≤ 0.001) (Table 4) existed only in the group of patients. In both patients and healthy participants, a positive correlation between the right and left hemispheres of the source LDAEP could be recorded. Regarding a relation between the serotonergic activity and the peripheral serotonin content, no relation was found in both groups. Additionally, the calculation of confounding factors, e.g., gender, smoking, marital status, and severity of depression revealed no effect on the serotonergic activity in both groups.

### 3.4. Genotyping of the 5-HTTLPR

Since 5-HTTLPR can be used as an indicator for an increased possibility of developing depression, we analyzed genotypes in both groups, to investigate a potential correlation between depression and alterations in this gene. The 5-HTTLPR polymorphism in the promoter region of the *SLC6A* has two different variants, a short allele (S) and a long allele (L). The s allele is associated with depression and lower gene transcription [32]. Since it is known that the s allele has greater susceptibility to depression it is called a risk allele. In agreement with other studies, the samples were divided into LL and S (LS + SS) genotypes (Table 5) [12,32]. There was no significant difference in the distribution between these two groups (Table 5). Genotype distributions were in Hardy–Weinberg equilibrium in the whole sample [χ^2^ = 0.658; *p* = 0.720]. The analysis did not reveal possible differences between the different genotypes of the 5-HTTLPR with symptoms of depression, serotonergic activity, and the peripheral serotonin content in both groups (Table 6).

The results concerning the 5HTTLPR genotypes should be interpreted with consideration of the small number of samples.

## 4. Discussion

The present study aimed to investigate the association of the peripheral serotonin content and the serotonergic activity in the CNS in patients with depression. In addition, the influence of the 5-HTTLPR on the serotonin content and serotonergic activation was examined. Moreover, the relation between the state of health and the serotonin content in the peripheral blood was assessed.

The results from the cortical LDAEP showed a tendency toward patients having lower serotonergic activity in CNS compared to healthy participants, although this difference was not statistically significant. These results are consistent with other studies showing no significant differences in serotonergic activity between the patients with major depression and healthy participants [22,33]. In contrast, the studies of Gallinat et al. (2000) and Ostermann et al. (2012) showed a significant difference between patients with depression and a healthy control group [34,35]. The inconsistency between these studies may be explained by possible factors that may influence the LDAEP, e.g., the administration of SSRIs or suicidal behavior [36,37,38]. Furthermore, gender, smoking, marital status, and the current severity of depression may affect the results of LDAEP [39]. In our study, gender, age, nicotine use, and marital status did not show any effect on LDAEP, whereas depression was associated with high LDAEP values. We found a correlation between the self-questionnaire STAI-X1 and cortical LDAEP only in the group of patients. Therefore, we suggest that a higher level of anxiety and depression correlates with higher values of LDAEP, which implicates a lower serotonergic activity. According to our knowledge, there is no other study describing a correlation between the LDAEP and anxiety in patients with depression. However, a positive correlation between LDAEP and anxiety has been found in patients with borderline personality disorder [40]. Moreover, two other studies revealed a relationship between the severity of depression and the cortical LDAEP [35,41]. The results suggest that the LDAEP value is related to anxiety and depression symptoms and might be a potential marker in patients with depression. Taken together, the serotonergic neurotransmission might be potentially associated with psychopathology (anxiety and depression). In addition, it is essential to consider all affecting and influencing factors on the serotonergic activity before making any conclusion on gained results. Here, future research is necessary to clarify the impact of the serotonergic activity, measured by LDAEP, on depression with careful consideration of possible confounding variables.

Furthermore, the present study reports a significant correlation between the cortical and source LDAEP in the group of patients but not in the control group. Contrary to these observations, Jaworska et al. (2012) suggested a correlation between all scalp-derived LDAEPs, in non-treated individuals with depression as well as in healthy participants. We could not replicate these findings for healthy participants [42]. In summary, the LDAEP results suggest an altered function of the serotonergic neurotransmission in patients with depression. In this study, the source and cortical LDAEP are comparable. Despite these results, the association of cortical and source LDAEP needs to be investigated and replicated in further studies. When extending the analysis to the serotonin content in the peripheral blood, no significant correlations between serotonin content and the LDAEP were observed. In contrast to our observations, Kawohl et al. (2008) reported a negative correlation between LDAEP and serum serotonin content in patients with affective disorders [24]. Since no other studies describe this correlation, we may speculate that this inconsistency is based on different designs of both studies. Further, it is necessary to investigate the association between the peripheral and the central serotonin systems. Regarding the content of serotonin in serum and platelets, we detected lower serotonin contents in patients compared to healthy participants. These findings are in line with several previous studies, whereas others revealed no differences in platelet serotonin content between patients suffering from depression and healthy participants [9,43,44,45]. Concerning the decreased peripheral serotonin concentration in patients compared to healthy controls, it should be considered that the influence of SSRI/SNRIs is reflected in these results. It cannot be assumed that patients with a depressive disorder generally have a reduced serotonin concentration. To make a more precise conclusion, unmedicated patients would have to be compared with healthy controls to exclude the influence of medication. Due to the small sample size of unmedicated patients (n = 10), this could not be investigated in this study.

Regarding these results, Saldanha and colleagues postulated that there is no direct correlation between serotonin levels and depression [46]. These results are consistent with our present findings since we did not find a correlation between the content of serotonin and the symptoms of depression in the group of patients.

In a previous study, the general influence of applied medication on patients with depression was observed [10]. Decreased serum serotonin contents were detected in patients who received SSRI/SNRI in general compared to patients without any psychotropic medications. These findings indicated the influence of SSRI/SNRI on the peripheral serotonin content. Previous studies have already proposed the influence of SSRIs on the serotonin content [8,9]. Further analysis showed that the application of other antidepressants excluding SSRI/SNRI did not affect the serum serotonin content in patients suffering from depression. The combination of SSRI/SNRI and other psychotropic medications revealed a good facility for treating depression. There are controversial results regarding the combination of psychotropic medications [47,48]. The assignment of various psychopharmacological medications can improve the personalized treatment of patients with depression and it is a possibility for treatment-resistant depression [48]. More investigations are adjuvant to clarify the implication of combining medications in the treatment of depression. Interestingly, Aleksovski and colleagues suggested that the peripheral abnormalities in serotonin content are caused by an altered function of the serotonin transporter [43,49].

However, we did not find any association between the altered serotonin transporter and the peripheral serotonin amount. The peripheral abnormalities of the serotonin content in patients may be caused by an altered function of the monoamine oxidase (MAO) or a deficient synthesis of serotonin. The result indicates an alteration in the peripheral serotonin synthesis that is not based on genetic variations in patients with depression. Further investigations are necessary to clarify if these abnormalities in the peripheral serotonin content are essential for the development of depression. Regarding the 5-HTTLPR genotype distribution, we did not find a different distribution of the 5-HTTLPR in patients and healthy participants. In contrast to our findings, Hoefgen et al. (2005) and Dorado et al. (2007) showed a significant difference in 5-HTTLPR distribution and claimed that the s-allele was significantly more highly distributed in patients suffering from depression in comparison to the healthy control group [50,51]. The contradictory results could be explained by a high difference in sample size (89 vs. 466).

When focusing on the association between 5-HTTLPR and depressive symptoms, a study by Laucht and colleagues (2009) reported higher rates of depression in LL-allele carriers in healthy individuals [52]. Other studies suggest an association between the short (s) allele distribution and increased susceptibility to depression and the stronger development of depressive symptoms [51,53,54]. Interestingly, Caspi et al. (2003) reported that stressful life events and childhood maltreatment might induce depression in participants carrying the less functional s-allele [54]. A meta-analysis supports the theory that the 5-HTTLPR s-allele moderates the relationship between stress and depression [55,56]. However, we did not assess environmental factors, and therefore we were not able to investigate differential susceptibility effects. In summary, our results are in accordance with current literature claiming no altered genotype distribution in individuals with depression. Furthermore, we cannot support an association between 5-HTTLPR and depression [57]. Influencing factors such as stress and environmental factors should be investigated in further studies, too.

In our study, no association between the distribution of the 5-HTTPLR and the serotonergic activity in the brain was found, despite previous findings reporting a potential correlation between the L/L genotype of the 5-HTTLPR and the LDAEP [58,59,60]. For instance, Gallinat et al. (2003) reported that individuals with an L/L genotype showed lower LDAEP [58]. In contrast, Strobel et al. (2003) indicated increased LDAEP in L/L individuals [59]. However, these highly contradictory results may be evoked by differences within the cohorts of both studies, including age, gender, or ethnic differences [60].

Despite a very careful design of this study considering cohort size, age, gender, and health state, we faced limitations, which might have affected our results. First, we would have to include more untreated patients in the study to clarify the influence of medication, e.g., SSRI, on the serotonergic activity in the brain and the serotonin levels in the peripheral blood. Second, the total sample size for the 5-HTTLPR genotyping should be higher. Hoefgen et al. (2005) emphasized a high sample size as a crucial factor for finding an association between certain indicators, e.g., 5-HTTLPR and depression [50]. Third, we should have measured the LDAEP at the follow-up again to see possible alterations in the serotonergic activity during the treatment. This is difficult to implement because it is time-consuming and most of the patients did not agree to participate in a second measurement. Lastly, we should have established additional questionnaires, which assess childhood trauma, stress, and suicidal tendencies of the participants, since previous studies postulated a possible association between depression and stress or suicidal behavior in patients with depression [38,55].

## 5. Conclusions

In conclusion, between the peripheral serotonin and the proposed marker of serotonergic activity, no association emerged.

Applied SSRI/SNRI medication showed an influence on the serotonin content in the serum of patients suffering from depression compared to patients with other psychotropic medications, or without psychotropic medication. Thus, further research is necessary to clarify the role of the components of the serotonergic system in the etiology of depression. It is essential to compare the serotonin contents from the cerebral spinal fluid (CSF) and the peripheral blood and investigate the role of serotonin in the development of depression.

## Figures and Tables

**Table 1:** Demographic, social, and clinical characteristics of healthy participants and patients with depression.

Variables	Healthy Controls	Patients with Depression
Number of subjects	89	89
Gender		
Male (n/%)	32 (36.0)	32 (36.0)
Female (n/%)	57 (64.0)	57 (64.0)
Age (mean ± SD)	37 (±12)	37 (±12)
Marital status		
single (n/%)	31 (34.8)	35 (39.3)
married (n/%)	27 (30.3)	25 (28.1)
in a partnership (n/%)	25 (28.1)	17 (19.1)
divorced (n/%)	6 (6.7)	1 (1.1)
widowed (n/%)	-	
Current patients status		
outpatient (n/%)	-	17 (19.1)
inpatient (n/%)	-	72 (80.9)
Ever in outpatient treatment before		
Yes (n/%)	-	56 (62.9)
No (n/%)	-	33 (37.1)
Age of first outpatient treatment (n = 52) (mean ± SD)	-	32 ± 14
Ever inpatient treatment before		
Yes (n/%)	-	72 (80.9)
No (n/%)	-	17 (19.1)
Age of first inpatient treatment (mean ± SD)	-	33 ± 12
Diagnosis		
Bipolar affective disorder (F31.3/F31.4) (n/%)	-	4 (4.5%)
Depressive episode (F32.0/F32.1/F32.2) (n/%)	-	34 (38.2%)
Recurrent depressive disorder (F33.0/F33.1/F33.2) (n/%)	-	51 (57.3%)
Medication		
Without psychotropic medication (n/%)	-	10 (11.2)
SSRIs/SNRIs + other psychotropic medication *^1^ (n/%)	-	57 (64.0)
Other psychotropic medications without SSRI/SNRI (n/%)	-	22 (24.7)
Only SSRIs/SNRIs (n/%)		20 (22.5)

*^1^ other psychotropic medication = antipsychotics, tricyclic antidepressant, tetracyclic antidepressant, lithium, benzodiazepine.

**Table 2:** Distribution of the antidepressant medications in patients.

Antidepressant	n (%)	Dosage Range (Min-Max)
SSRI		
Escitalopram/cipralex	7 (7.9)	10–20 mg/day
Citalopram	9 (10.1)	10–40 mg/day
Sertraline	13 (14.6)	50–150 mg/day
Elontrile/bupropion	6 (6.7)	100–300 mg/day
Fluoxetine	2 (2.2.)	25–30 mg/day
Trazodone	1 (1.1.)	100 mg/day
SNRI		
Venlaflaxine/trevilor	15 (16.9)	50–375 mg/day
Milnacipran/milnaneurax	2 (2.2)	12.5–25 mg/day
Duloxetine/cymbalta	4 (4.5)	90–120 mg/day
Tetracyclic antidepressants		
Mirtazapine	13 (14.6)	6.5–30 mg/day
Tricyclic antidepressant		
Trimipramine/trimineurine	9 (10.1)	25–100 mg/day
Opipramole	1 (1.1)	300 mg/day
Amitriptyline/amitrile	2 (2.2)	25–75 mg/day
Doxepin	2 (2.2)	50–150 mg
Melatonin receptor agnonist		
Valdoxan	6 (6.7)	25–50 mg
Total number of patients receiving antidepressants	69 (77.5)	

**Table 3:** Psychometric data, serotonin content (serum/platelet), and serotonergic activity of healthy participants and patients with depression.

Variables	Healthy Controls	Patients with Depression	Statistics
Total number of subjects	89	89	
Psychometry			
HAMD-21 ^a^ (mean ± *SD*)	1.48 ± 2.00	18.56 ± 5.21	*t*(176) = 28.9, *p* < 0.001 ***
BDI-II ^d^ (mean ± *SD*)	3.83 ± 5.12	33.25 ± 10.46	*t*(176) = 23.8, *p* < 0.001 ***
STAI-X1 ^b^ (mean ± *SD*)	33.33 ± 7.98	55.36 ± 12.34	*t*(176) = 14.2, *p* < 0.001 ***
STAI-X2 ^c^ (mean ± *SD*)	32.64 ± 7.20	60.56 ± 9.20	*t*(176) = 22.6, *p* < 0.001 ***
Serotonin content			
Platelet serotonin content (10^9^/Thr) (mean ± *SD*) (n = 71)	2008.8 ± 1026.4	1113.9 ± 964.1	*t*(142) = −5.4, *p* < 0.001 ***
Serum serotonin content (ng/mL) (mean ± *SD*) (n = 87)	773.8 ± 646.0	230.9 ± 326.0	*t*(172) = −6.1, *p* < 0.001 ***
Serotonergic activity			
Cortical LDAEP (mean ± *SD*) (n = 66)	0.252 ± 0.119	0.256 ± 0.186	*t*(130) = 0.15, *p* = 0.855
Source LDAEP (left) (mean ± *SD*) (n = 66)	0.269 ± 0.512	0.207 ± 0.204	*t*(130) = −0.92, *p* = 0.362
Source LDAEP (right) (mean ± *SD*) (n = 66)	0.253 ± 0.498	0.191 ± 0.245	*t*(130) = −0.90, *p* = 0.371

*** *p* < 0.001; ^a^ Hamilton = HAMD-21; ^b^ State Anxiety Inventory = STAI-X1; ^c^ Trait Anxiety Inventory = STAI-X2; ^d^ Beck Depression Inventory = BDI-II.

**Table 4:** Correlation between the patients with depression and healthy participants. The first section summarizes the correlation with the serotonin content in platelet, the second section shows the correlations between cortical LDAEP, STAI-X1, BDI-II, and source LDAEP and the lowest section contains the correlation of the source LDAEP. Results reported are Pearson correlation coefficient r with significant correlations printed in bold and * indicating results with * *p* < 0.05, ** *p* < 0.01 and representing *** *p* < 0.001. The correlations within the self-questionnaires are not shown.

	n	Patients with Depression	Healthy Participants
		Platelet serotonin content	
Serum serotonin content	72	*r* = −0.050; *p* = 0.676	*r* = 0.350; *p* = 0.003 **
STAI-X2	72	*r* = 0.170; *p* = 0.154	*r* = −0.236; *p* = 0.046 *
HAMD-21	72	*r* = −0.068; *p* = 0.569	*r* = −0.240; *p* = 0.042 *
		Cortical LDAEP	
STAI-X1	66	*r* = 0.257; *p* = 0.037 *	*r* = −0.133; *p* = 0.368
BDI-II	66	*r* = 0.258; *p* = 0.036 *	*r* = −0.087; *p* = 0.485
Source LDAEP (right hemisphere)	66	*r* = 0.488; *p* < 0.001 ***	*r* = −0.015; *p* = 0.908
Source LDAEP (left hemisphere)	66	*r* = 0.633; *p* < 0.001 ***	*r* = −0.001; *p* = 0.994
		Source LDAEP (right hemisphere)	
Source LDAEP (left hemisphere)	66	*r* = 0.454; *p* < 0.001 ***	*r* = 0.934; *p* < 0.001 ***

**Table 5:** Distribution of the 5-HTTLPR.

	5-HTTLPR Genotypes				
	**LL**	**LS**	**SS**	**L (LL)**	**S (LS + SS)**
Patients with depression (n/%)	32 (35.96)	36 (40.45)	21 (23.60)	32 (37.1)	57 (62.9)
Healthy participants (n/%)	27 (30.34)	40 (44.94)	22 (24.72)	27 (39.3)	62 (69.7)

**Table 6:** Differences between genotypes concerning the psychometry, peripheral serotonin content, and serotonergic activity in patients and healthy participants.

	Patients		Healthy Participants	
	**LL**	**LS/SS**	**LL**	**LS/SS**
Psychometry				
HAMD-21 ^a^ (mean ± *SD*) (n)	17.94 ± 6.60 (32)	18.91 ± 4.27 (57)	1.50 ± 1.88 (26)	1.48 ± 2.06 (63)
*t*-test	*t*(45.87) = 0.753; *p* = 0.456		*t*(87) = −0.051; *p* = 0.960	
BDI-II ^d^ (mean ± *SD*)	34.53 ± 10.05 (32)	32.53 ± 10.70 (57)	3.27 ± 3.04 (26)	4.06 ± 5.76 (63)
*t*-test	*t*(87) = −0.867; *p* = 0.389		*t*(87) = 0.664; *p* = 0.508	
STAI-X1 ^b^ (mean ± *SD*)	54.56 ± 12.87 (32)	55.81 ± 12.12 (57)	31.88 ± 6.49 (26)	33.92 ± 8.49 (63)
*t*-test	*t*(87) = 0.455; *p* = 0.651		*t*(87) = 1.10; *p* = 0.276	
STAI-X2 ^c^ (mean ± *SD*)	59.75 ± 11.12 (32)	61.02 ± 7.99 (57)	31.69 ± 6.73 (26)	33.03 ± 7.40 (63)
*t*-test	*t*(49.32) = 0.568; *p* = 0.573		*t*(87) = 0.797; *p* = 0.428	
Serotonin content				
Platelet serotonin content(10^9^/Thr) (mean ± *SD*) (n)	1139.26 ± 994.50 (27)	1098.65 ± 956.40 (45)	2164.42 ± 1016.54 (22)	1940.30 ± 1033.37 (50)
*t*-test	*t*(70) = −0.172; *p* = 0.864		*t*(70) = −0.852; *p* = 0.397	
Serum serotonin content(ng/mL) (mean ± *SD*) (n)	252.54 ± 354.20 (32)	231.10 ± 323.11 (56)	734.77 ± 594.87 (26)	788.03 ± 665.39 (62)
*t*-test	*t*(86) = −0.289; *p* = 0.773		*t*(86) = 0.353; *p* = 0.725	
Serotonergic activity				
Cortical LDAEP (mean ± *SD*) (n)	0.278 ± 187 (24)	0.242 ± 0.186 (42)	0.234 ± 0.132 (19)	0.256 ± 0.116 (47)
*t*-test	*t*(64) = −0.744; *p* = 0.459		*t*(64) = 0.690; *p* = 0.496	
Source LDAEP (left) (mean ± *SD*) (n)	0.217 ± 0.195 (24)	0.201 ± 0.209 (42)	0.211 ± 0.145 (19)	0.299 ± 0.601 (47)
*t*-test	*t*(64) = −0.299; *p* = 0.766		*t*(64) = 0.630; *p* = 0.531	
Source LDAEP (right) (mean ± *SD*) (n)	0.227 ± 0.250 (24)	0.171 ± 0.243 (42)	0.177 ± 0.255 (19)	0.284 ± 0.568 (47)
*t*-test	*t*(64) = −0.894; *p* = 0.374		*t*(64) = 0.789; *p* = 0.433	

^a^ Hamilton = HAMD-21; ^b^ State Anxiety Inventory = STAI-X1; ^c^ Trait Anxiety Inventory = STAI-X2; ^d^ Beck Depression Inventory = BDI-II.

## Data Availability

The data presented in this study are available in Supplementary Materials Tables S1–S3 and Figure S1.

## Supplementary Materials

The following supporting information can be downloaded at: https://www.mdpi.com/article/10.3390/bs12060178/s1, Table S1: Data from the serotonin content (platelet/serum), LDAEP (cortical/source), psychometric questionnaires (HAMD-21, BDI-II, STAI-X1, STAI-X2) and genotyping of all participants, Table S2: Derivation of applied medication of patient with depression, Table S3: Comparison of patients receiving no psychotropic medication with patients who receiving psychotropic medications; Figure S1. Correlations of the peripheral 5-HT content, serotonergic activity, and symptoms of anxiety/depression in patients and healthy participants. A Correlation between platelet 5-HT content and serum 5-HT content in healthy participants. B Correlation between the STAI-X2 and platelet 5-HT content in healthy participants. C Correlation between the HAMD-21 and platelet 5-HT content in healthy participants. D Correlation between the STAI-X1 and the cortical LDAEP in patients. E Correlation between the BDI-II and cortical LDAEP in patients. F Correlation between source LDAEP (left hemisphere) and cortical LDAEP in patients. G Correlation between source LDAEP (right hemisphere) and cortical LDAEP in patients. H Correlation between the source LDAEP (left hemisphere) and source LDAEP (right hemisphere) in patients. I Correlation between the source LDAEP (left hemisphere) and source LDAEP (right hemisphere) in healthy participants. r = Pearson correlations coefficient
