# Supplementary material for: Investigation of the Serotonergic Activity and the Serotonin Content in Serum and Platelet, and the Possible Role of the Serotonin Transporter in Patients with Depression
Source: Behav Sci (Basel). 2022 Jun 3;12(6):178. doi: 10.3390/bs12060178 (PMC9220674; doi:10.3390/bs12060178)
Supplement: Supplementary file 1 [file behavsci-12-00178-s001.zip › Supporting Information Figure S1.pdf]

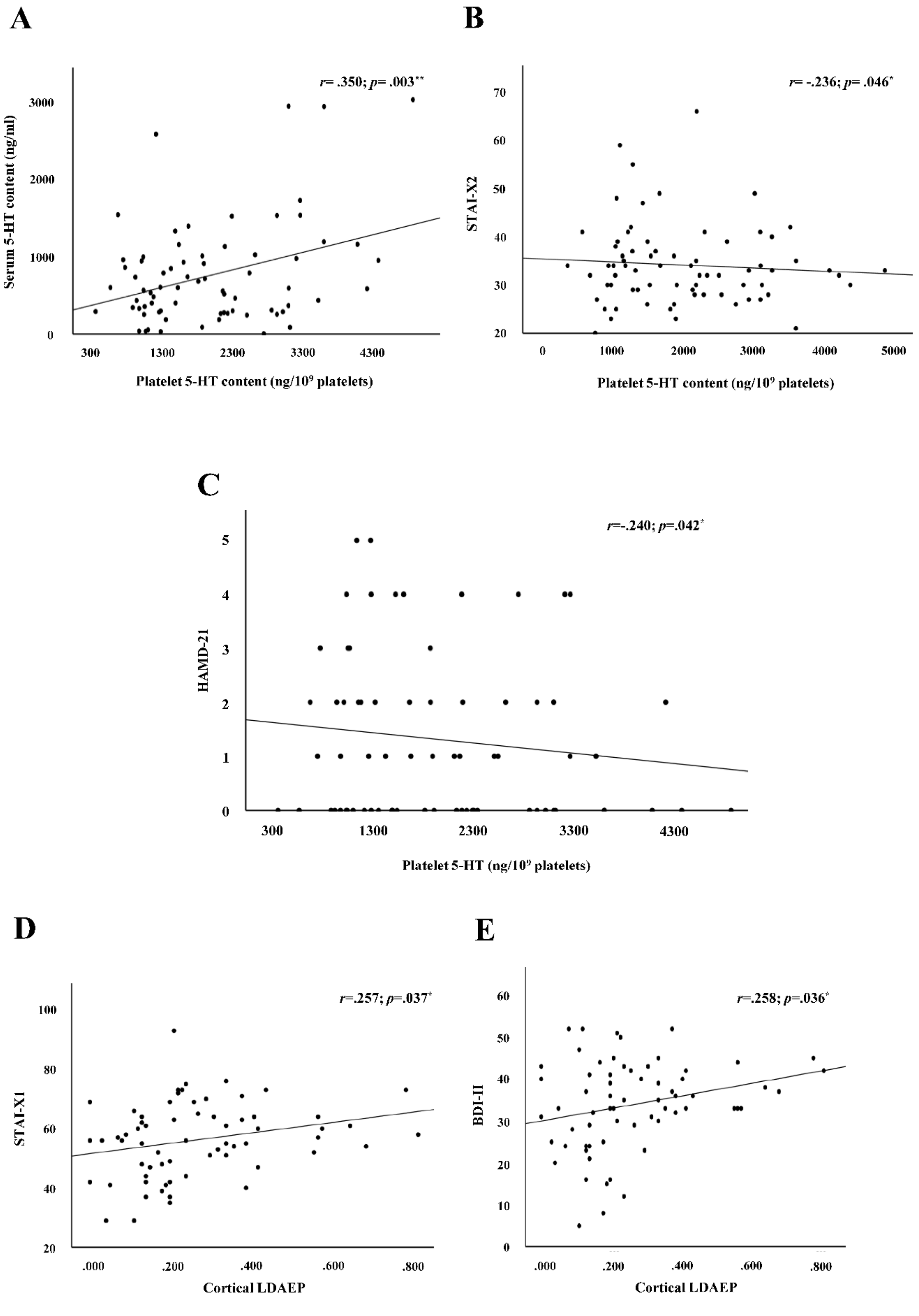
A
r= .350; p= .003**
Serum 5-HT content (ng/ml)
Platelet 5-HT content (ng/10⁹ platelets)
B
r= -.236; p= .046*
STAI-X2
Platelet 5-HT content (ng/10⁹ platelets)
C
r=-.240; p=.042*
HAMD-21
Platelet 5-HT (ng/10⁹ platelets)
D
r=.257; p=.037*
STAI-X1
Cortical LDAEP
E
r=.258; p=.036*
BDI-II
Cortical LDAEP

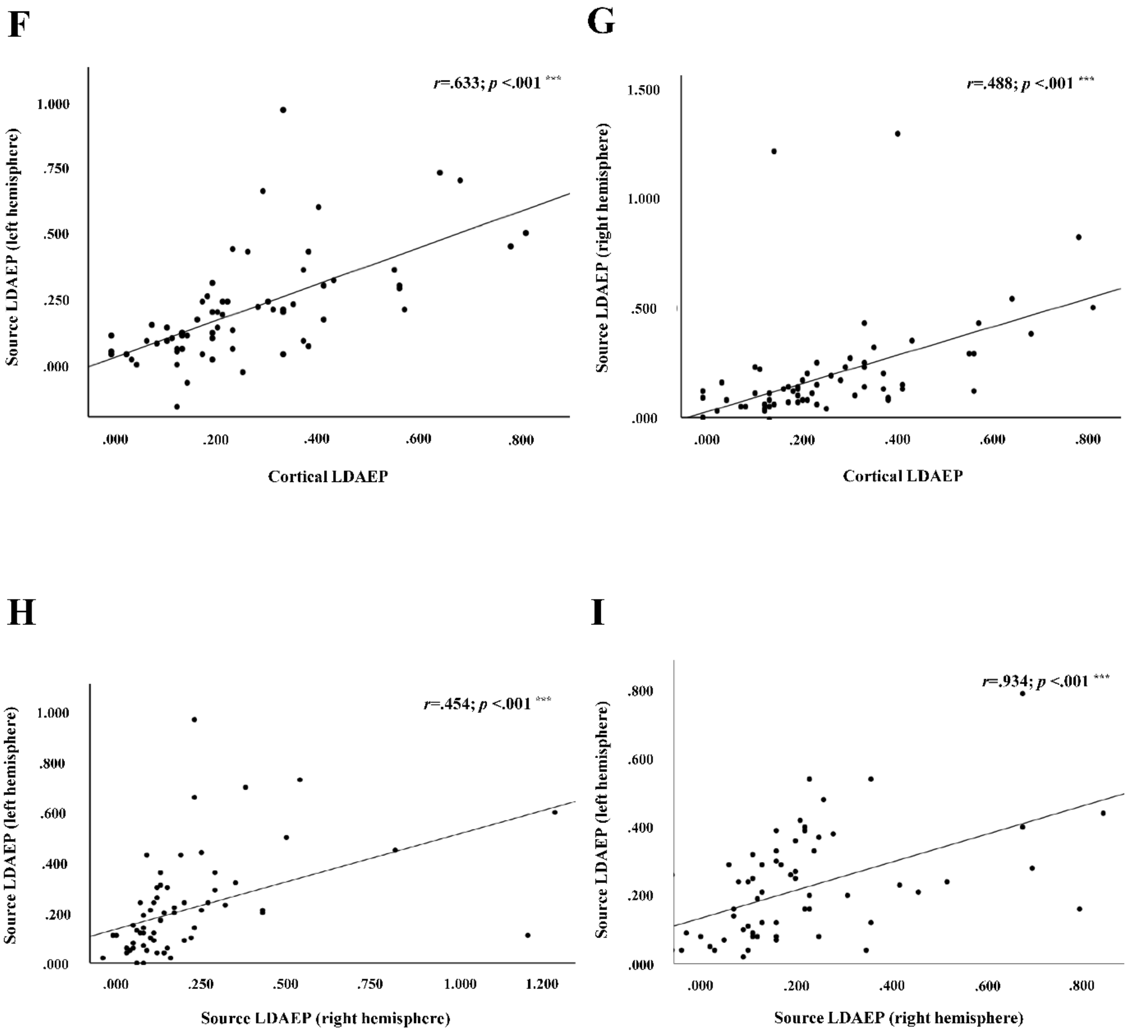

Figure S1. Correlations of the peripheral 5-HT content, serotonergic activity, and symptoms of anxiety/depression in patients and healthy participants. A Correlation between platelet 5-HT content and serum 5-HT content in healthy participants. B Correlation between the STAI-X2 and platelet 5-HT content in healthy participants. C Correlation between the HAMD-21 and platelet 5-HT content in healthy participants. D Correlation between the STAI-X1 and the cortical LDAEP in patients. E Correlation between the BDI-II and cortical LDAEP in patients. F Correlation between source LDAEP (left hemisphere) and cortical LDAEP in patients. G Correlation between source LDAEP (right hemisphere) and cortical LDAEP in patients. H Correlation between the source LDAEP (left hemisphere) and source LDAEP (right hemisphere) in patients. I Correlation between the source LDAEP (left hemisphere) and source LDAEP (right hemisphere) in healthy participants. r = Pearson correlations coefficient.
