# Supplementary material for: Investigation of the Serotonergic Activity and the Serotonin Content in Serum and Platelet, and the Possible Role of the Serotonin Transporter in Patients with Depression
Source: Behav Sci (Basel). 2022 Jun 3;12(6):178. doi: 10.3390/bs12060178 (PMC9220674; doi:10.3390/bs12060178)
Supplement: Supplementary file 1 [file behavsci-12-00178-s001.zip › Supporting Information Table S1.pdf]

Table S1. Data from the serotonin content (platelet/serum), LDAEP (cortical/source), psychometric questionnaires (HAMD-21, BDI-II, STAI-X1, STAI-X2), and genotyping of all participants.

Participant ID	Platelet serotonin (10^9/Thr)	Serum serotonin (ng/ml)	Cortical LDAEP	Source LDAEP (left)	Source LDAEP (right)	HAMD-21	BDI-II	STAI-X1	STAI-X2	5-HTTLPR genotype
P001	53327.46*	14.89	0.29	0.66	0.23	8	23	51	52	LS
P002	41.62*	19.43	0.19	0.31	0.13	0	39	49	57	LL
P003	60124.45*	39.7	0.26	0.43	0.19	17	29	65	63	LL
P004	1801.23	631.95	0.2	0.2	0.17	13	45	93	77	SS
P005	625.32	223.69	0.43	0.32	0.35	24	36	73	63	LS
P006	227.75*	1071.04	0.13*	0.12*	0.08*	24	29	37	47	LL
P007	1376.55	638.77	0.57	0.21	0.43	18	33	60	61	LL
P008	217.34*	68.49	0.4	0.6	1.29	22	40	64	71	LS
P009	961.4	857.42	0.04*	-0.02*	0.03*	23	38	47	60	SS
P010	903.43	46.29	0.37	0.09	0.2	19	37	63	58	SS
P011	714.83*	67.8	0.28*	0.36*	0.08*	16	36	45	58	LS
P012	1144.28	1255.61	0.52*	0.37*	0.22*	14	31	55	48	LL
P013	907.18	116.21	0.12	0.05	0.04	13	23	48	46	SS
P014	1361.95	836.35	0.37	0.36	0.13	31	52	71	70	LS
P015	174.29	350.44	0.78*	0.45*	0.82*	21	45	73	65	LL
P016	1027.34	17.69	0.12	-0.16	0.06	20	37	62	64	SS
P017	129.85	186.16	0.21	0.24	0.2	22	51	73	75	LL
P018	859.58	128.37	0.25	-0.03	0.04	24	42	69	67	LL
P019	129.85	74.32	-	-	-	12	23	51	54	LL
P020	521.3*	942.8*	0.2*	1.3*	0.17*	23	33	58	52	LS
P021	204.64	1186.09	0.33	0.2	0.43	18	39	61	69	SS
P022	1467.49	559.4	0.12	0.06	0.03	3	24	55	66	LL
P023	1027.34	111.49	0.41	0.17	0.13	20	33	60	57	LS
P024	1172.64	829.91	0.12*	0	0.06*	15	16	64	65	LS
P025	714.83	715.78	0.55	0.36	0.29	15	33	52	63	LL
P026	870.34	1276.61	0.56	0.29	0.29	20	33	64	69	LS

P027	1431.43	48.45	0.07*	0.13*	-0.02*	11	17	58	57	SS
P028	-	451.59	0.21	0.19	0.08	20	30	72	69	LS
P029	276.51	73.69	0.68	0.7	0.38	24	37	54	63	LL
P030	1879.06	31.95	0.23	0.44	0.25	15	12	44	55	LS
P031	2228.6	243.95	0.36*	0.17*	0.04*	23	46	71	63	LL
P032	1695.33	168.45	0.07	0.15	0.05	24	52	56	68	LS
P033	3654.1	11.57	0.38*	0.07*	0.08*	22	36	55	57	LL
P034	536.16*	52.27	-	-	-	12	6	31	45	SS
P035	271.01	84.86	0.18*	0.2*	0.19*	19	35	60	58	LL
P036	1823.42	250.26	0.41	0.3	0.15	18	42	47	68	LL
P037	3.06	10.65	0.1	0.14	0.23	20	47	66	74	LL
P038	1335.63	38.86	0.16*	0.12*	0.1*	10	29	41	58	LL
P039	525.46	64.58	0.64	0.73	0.54	20	38	61	54	LL
P040	1212.52	1093.12	0.31	0.21	0.1	19	31	53	55	LS
P041	40586.16*	13.16	-	-	-	24	24	56	54	SS
P042	1450.64	239.46	0.1*	0.09*	0.07*	19	25	40	48	LS
P043	4861.2	11.2	0.11	0.1	0.22	14	52	60	71	LS
P044	210.19	254.11	0.3	0.24	0.27	21	43	64	70	LL
P045	174.29	38.68	0.22	0.24	0.11	19	50	73	57	SS
P046	11588.33*	9.37	0.23	0.13	0.06	22	43	75	69	SS
P047	1534.12	214.11	0.03	0.02	0.16	15	20	29	56	LL
P048	1245.4	30.9	-0.01	0.05	0.09	28	43	56	66	LL
P049	342.5	30.43	0.2	0.14	0.08	21	33	63	70	LL
P050	3214.31	9.1	0.19	0.1	0.1	22	41	69	71	LL
P051	143.56	159.74	-	-	-	22	30	56	62	LS
P052	3099.19	18.82	0.33	0.21	0.25	16	45	76	77	LL
P053	588.79	106.21	0.13	0.12	0.11	19	24	61	54	LS
P054	467.11	130.77	0.13*	0.06*	0.05*	7	21	44	64	LS
P055	1744.28	66.55	0.1*	0.14*	0.19*	17	36	43	66	LL
P056	1077.32	119.47	0.56	0.3	0.12	23	44	57	65	SS
P057	529.03	74.51	0.61*	0.08*	0.04*	18	40	61	76	SS

P058	4761.68	12.67	-0.01	0.04	0.12	19	31	69	60	SS
P059	1146.17	124.5	-	-	-	19	17	48	46	LS
P060	-	256.57	0.28	0.22	0.17	14	40	70	66	LS
P061	-	125.8	0.06	0.09	-0.32	21	24	57	65	SS
P062	-	16.01	0.14	0.11	1.21	21	32	47	43	LL
P063	1875.02	515.86	0.04	0	0.08	17	33	41	60	LS
P064	-	-	0.18	0.26	0.12	24	15	41	51	LS
P065	-	970	0.1	0.09	0.11	6	5	29	31	LL
P066	1071.64	350.46	-	-	-	23	22	70	61	LS
P067	536.63	82.76	0.28*	0.17*	0.12*	24	41	44	62	LS
P068	1600.69*	126.68	0.81	0.5	0.5	14	42	58	69	SS
P069	434.9	563.97	0.14	-0.07	0.06	23	32	47	52	LS
P070	2013.18	511.19	0.13	0.11	-0.01	26	41	42	62	LS
P071	525.46	212.66	0.23	0.06	0.15	18	35	56	67	LS
P072	767.11	9.36	0.57*	0.25*	0.32*	18	31	33	38	LL
P073	272.73	5.27	0.33	0.97	0.23	20	35	51	67	LS
P074	626.94	6.02	0.19	0.2	0.14	17	33	35	62	LS
P075	427.64	5.97	0.17	0.24	0.07	20	8	48	57	LS
P076	416.99	20.63	0.38*	0.43*	0.09*	16	32	40	38	LS
P077	295.41	5.59	0.19	0.12	0.07	18	36	42	57	LS
P078	61.58	6.46	0.35	0.23	0.32	16	33	54	49	LL
P079	1856.97	8.16	0.47*	0.62*	0.56*	22	39	74	72	LS
P080	1209.87	97.66	-0.01	0.11	0	19	40	42	63	LS
P081	1229.59	16.51	0.33	0.04	0.14	15	30	55	61	SS
P082	791.45	34.64	0.17	0.04	0.14	19	25	39	56	SS
P083	667.88	20.52	0.14	0.14	0.22	21	42	53	64	SS
P084	830.79	10.97	0.08*	0.08*	0.05*	27	28	58	65	LS
P085	766.27	236.66	0.16	0.17	0.13	17	44	52	68	LS
P086	657.17	122.53	0.19*	0.02*	-0.04*	15	16	37	44	LL
P087	582.13	29.52	0.17*	0.06*	0.07*	22	31	54	65	LL
P088	681.52	44.4	-	-	-	25	49	70	71	SS

P089	487.29	55.36	0.02	0.04	0.03	17	25	56	61	SS
HP001	1143.57	538.95	0.49*	0.66*	0.46*	5	6	36	34	LL
HP002	1051.36	255.17	0.22	0.25	0.11	0	1	25	25	SS
HP003	1530.36	600.34	0.47	0.33	0.16	4	3	30	32	SS
HP004	2551.39	791.88	0.13	0.2	0.31	1	0	28	32	LS
HP005	780.37	864.32	0.28	0.16	0.07	3	0	27	26	LS
HP006	926.8	737.51	0.26	0.4	0.22	0	6	30	33	LL
HP007	1822.78	683.13	0.29	0.08	0.25	0	1	25	22	SS
HP008	1039.38	1001.9	0.45	0.24	0.1	0	5	32	36	LS
HP009	-	-	-	-	-	0	0	36	32	SS
HP010	1043.36	567.18	0.37	0.27	0.2	4	7	38	34	LL
HP011	1682.22	1398.81	0.29*	0.34*	0.14*	1	4	34	40	LS
HP012	1901.03	915.16	0.48	0.23	0.42	1	0	23	28	LS
HP013	1546.57	1161.38	0.53	0.04	0.35	0	1	36	31	SS
HP014	-	561.01	0.06	0.11	0.1	1	4	35	32	SS
HP016	1015.82	-	0.37*	0.28*	0.4*	2	3	34	34	LS
HP018	1059.43	948.03	0.13*	0.57*	0.37*	3	15	48	49	LS
HP019	1612.97	1324.34	0.77	1.39	1.24	4	0	37	32	LS
HP020	1221.53*	933.12	0.18	0.21	0.46	0	0	41	35	LS
HP021	3036.91*	1219.28	0.01	0.09	-0.16	4	2	23	25	LL
HP023	1494.29	-	0.38	0.44	0.85	0	1	26	30	LL
HP024	-	1335.98	0.32*	0.25*	0.14*	0	3	34	35	LS
HP025	-	1346.25	0.41	0.26	0.19	0	0	25	31	LL
HP026	-	324.24	0.25*	0.19*	0.12*	0	0	27	23	SS
HP029	-	-	0.18	0.04	0.1	7	11	37	40	SS
HP031	-	-	0.24*	-0.02*	-0.01*	0	0	22	22	LS
HP032	-	2040.33	-	-	-	6	9	34	53	LL
HP033	3612.44	657.47	0.39	0.16	0.8	0	0	35	32	LL
HP035	1916.76	-	0.09*	0.09*	-0.03*	0	0	30	29	LL
HP036	1432.78	717.87	0.34	0.42	0.21	1	6	47	37	SS
HP037	4224.82	850.25	0.22	0.2	0.23	2	0	32	35	SS

HP039	2630.6	1970.58	0.09	0.07	0.16	2	7	39	37	LL
HP041	571.8	1029.68	-	-	-	0	6	41	42	LL
HP042	2299.59	855.56	0.23	0.3	0.16	0	1	28	28	SS
HP044	1288.39	1527.13	0.4	0.33	0.24	4	3	55	47	LS
HP045	1288.68	30.72	0.07*	0.05*	0.04*	0	0	29	29	LS
HP046	688.01*	302.31	0.21	0.08	0	0	0	25	23	SS
HP047	980.69	37.71	0.41	0.48	0.26	0	0	23	27	LL
HP048	2118.64	188.11	0.1	0.02	0.09	1	9	34	34	SS
HP049	848.29*	801.01	0.42	0.24	0.52	0	6	32	38	LS
HP050	680.13	1546.4	-0.04*	0.16*	0.09*	2	3	32	30	SS
HP051	3001.04*	226.65	0.18	0.12	0.13	0	2	29	27	LL
HP052	2942.81	1536.85	-	-	-	0	0	27	23	LL
HP053	3609.97	1197.95	0.27*	0.32*	0.11*	0	0	21	21	LL
HP054	3127.54	87.06	0.42*	0.85*	0.75*	0	0	30	26	LL
HP055	754.44	964.8	0.44	0.24	0.08	1	0	20	20	SS
HP056	3495.62*	1423.98	0.24	0.16	0.22	1	5	23	33	SS
HP057	3270.79	1730.99	0.24*	0.08*	0.09*	1	8	40	37	LS
HP058	3273.08	1540.08	0.28	0.14	0.07	4	6	33	32	LS
HP059	4088.83	1164.89	0.25	0.13	0.07	0	10	33	27	LS
HP060	3026.35	291.03	0.15	0.04	-0.06	0	2	49	27	LL
HP061	2755.47	4.18	0.43	0.39	0.22	4	12	26	39	SS
HP062	3530.03	440.09	0.18*	0.07*	0.05*	1	2	42	29	LL
HP063	3110.08	593.36	-	-	-	2	19	34	30	LS
HP064	4385.21	956.27	0.29	0.08	0.12	0	1	30	29	LS
HP065	3107.9	2946.23	0.12	0.04	0.03	0	0	27	28	LS
HP066	4877.36	3029.86	0.18	0.06	0.12	0	4	33	36	LS
HP067	2140.34	266.84	0.26	0.09	0.11	0	0	29	30	LS
HP068	9689.9*	636.36	0.24	0.26	-0.06	1	7	31	29	LS
HP069	3218.57	981.35	0.41	0.29	0.06	4	5	28	32	LL
HP070	2311.61	303.72	0.27	0.25	0.2	0	10	41	37	LS
HP071	3105.23	371.49	0.27	-0.02	-0.08	2	6	41	37	LS

HP072	1993.56*	710.27	0.27*	0.23*	0.05*	4	9	33	47	LS
HP073	2191.08	522.56	0.24*	0.37*	0.25*	0	0	35	27	LS
HP074	2189.39	279.76	0.14	0.36	0.2	4	8	30	30	LL
HP075	2170.98	557.52	0.3	0.16	0.23	1	5	28	39	LL
HP076	1498.69	404.9	0.49*	-0.18*	0.76*	0	6	39	35	LS
HP077	1879.08	1014.11	0.29	0.38	0.28	2	0	26	30	LS
HP078	4282.44*	221.73	0.16	0.08	0.16	1	1	34	27	LS
HP079	1160.47	404.11	0.2	0.05	0.02	2	2	35	32	LL
HP080	2865.75	312.11	0.36*	0.31*	0.48*	0	1	30	28	SS
HP081	1108.2	54.86	0.15	0.08	0.11	0	5	59	36	LL
HP082	981.94	334.28	0.18	0.28	0.7	1	5	30	29	LL
HP083	360.68	293.87	0.19	0.4	0.68	12	35	34	59	LL
HP084	2942.7	257.17	0.13	0.29	0.17	2	6	33	31	SS
HP085	1073.85	35.76	0.14	4.17	3.9	3	10	39	40	LS
HP086	1876.92	89.16	0.22	0.12	0.16	3	0	36	30	LS
HP087	2346.99	468.4	0.14	0.12	0.36	0	0	32	37	LS
HP088	2514.97	246.73	-	-	-	1	0	32	21	SS
HP089	1360.97	187.99	0.28	0.79	0.68	0	4	29	30	SS
HP090	1326.79	791.4	0.08	0.04	-0.04	2	7	33	42	LL
HP091	890.11	346.66	-	-	-	0	0	25	28	LS
HP092	2240.36	270.69	0.28	0.39	0.16	0	0	32	26	LS
HP093	945.45	438.57	0.2	0.1	0.09	2	0	34	35	LS
HP094	1283.45	605.53	0.33	0.21	0.13	5	3	37	35	SS
HP095	1185.61	486.81	0.37*	0.23*	0.22*	2	1	34	30	LL
HP096	1669.47	741.55	0.03	-0.1	0.07	2	2	49	45	LS
HP097	1113.19*	1183.66	0.06	0.54	0.23	1	0	28	26	LS
HP098	1264.46	287.13	0.31	0.29	0.13	1	4	42	38	LS
HP100	2199.74	1136.49	0.35	0.54	0.36	2	5	66	49	LS

* Values are not included in the calculations. Reason for excluding these values were missing data of the matched pair, outliers or the values did not achieve the criteria for further analysis (for example LDAEP analysis).
