# Supplementary material for: Investigation of the Serotonergic Activity and the Serotonin Content in Serum and Platelet, and the Possible Role of the Serotonin Transporter in Patients with Depression
Source: Behav Sci (Basel). 2022 Jun 3;12(6):178. doi: 10.3390/bs12060178 (PMC9220674; doi:10.3390/bs12060178)
Supplement: Supplementary file 1 [file behavsci-12-00178-s001.zip › Supporting Information Table S2.pdf]

Table S2. Derivation of applied medication of patient with depression.

**Medication**	**Patients with depression**
Number of subjects	89
**Medication**	
Only SSRIs/SNRIs (n/%)	20 (22.5)
Only antipsychotics (n/%)	8 (9.0)
Only other antidepressants*[1] (n/%)	6 (6.7)
SSRIs/SNRIs + antipsychotics (n/%)	18 (20.2)
SSRIs/SNRIs + other antidepressants* (n/%)	9 (10.1)
SSRIs/SNRIs + other antidepressants* + antipsychotics (n/%)	13 (14.6)
Lithium (n/%)	2 (2.2)
Benzodiazepine (n/%)	5 (5.6)
Melatonin receptor antagonist (n/%)	6 (6.7)
Z-Substance (n/%)	2 (2.2)
Anticonvulsants (n/%)	2 (2.2)
Herbal antidepressants (n/%)	1 (1.1)

*[1] other antidepressants= tricyclic- and tetracyclic antidepressants.
