# Supplementary material for: Investigation of the Serotonergic Activity and the Serotonin Content in Serum and Platelet, and the Possible Role of the Serotonin Transporter in Patients with Depression
Source: Behav Sci (Basel). 2022 Jun 3;12(6):178. doi: 10.3390/bs12060178 (PMC9220674; doi:10.3390/bs12060178)
Supplement: Supplementary file 1 [file behavsci-12-00178-s001.zip › Supporting Information Table S3.pdf]

Table S3. Comparison of patients receiving no psychotropic medication with patients who receive psychotropic medications.

	Without medication	SSRIs/SNRIs +other psychotropic medication	Other psychotropic medication, without SSRIs/SNRIs	Only SSRIs/SNRIs	Only other AD*	Only antipsychotics	SSRIs/SNRIs + other AD*	SSRIs/SNRIs + antipsychotics	SSRIs/SNRIs + other AD + antipsychotic s
Serum serotonin (ng/ml) (n)	585.49 ± 501.28 (10)	135.20 ±241.27 (56)	345.32 ± 318.10 (22)	111.10 ± 187.19 (20)	384.44 ± 348.19 (6)	362.14 ± 371.64 (8)	127.58 ± 264.69 (9)	195.88 ± 336.64 (18)	221.08 ± 262.82 (13)
t-test		*t*(64)=4.49, *p* <.001***	*t*(30)= 1.65 *p*=.110	*t*(10.26)=2.89 , *p*=.016*	*t*(14)=.860, *p*=.404	*t*(16)=1.52, *p*=.148	*t*(17)=2.45, *p*=.026	*t*(25)=2.42, *p*=.023*	*t*(21)=2.26, *p*=.035*
Platelet serotonin (10^9/Thr) (n)	655.61 ± 369.36 (7)	1123.48 ± 1081.41 (53)	962.65 ± 609.75 (20)	952.54 ± 828.73 (20)	857.98 ± 549.82 (5)	991.23 ± 671.07 (8)	1554.04 ± 1384.0 (9)	1044.91 ± 1014.88 (18)	1441.88 ± 1214.14 (12)
t-test		*t*(57)=-1.05 *p*=.300	*t*(24)=-1.16 *p*=.257	*t*(24)=-.845 *p*=.407	*t*(9)=-.729, *p*=.485	*t*(13)=-1.03, *p*=.323	*t*(12)=-1.53, *p*=-.151	*t*(20)=-.905, *p*=.376	*t*(16)=-1.53, *p*=.145
HAMD-21 (n)	16.00 ± 5.72 (10)	18.53 ± 5.12 (57)	19.82± 5.00 (22)	18.10 ± 5.17 (20)	20.00 ± 2.00 (6)	15.75 ±5.80 (8)	17.00 ± 5.431 (9)	18.61 ± 6.06 (18)	21.54 ± 3.89 (13)
t-test		*t*(65)=-1.41 *p*= .162	*t*(30)=-1.92 *p*=.064	*t*(28)=-.101, *p*= .320	*t*(14)=1.64, *p*=.124	*t*(16)=.665, *p*=.515	*t*(17)=-.390, *p*=.702	*t*(26)=-1.11, *p*=.275	*t*(21)=-2.77, ***p***=.012*
BDI-II (n)	31.40 ± 11.38 (10)	33.07 ± 10.72 (57)	34.55 ± 9.65 (22)	29.05 ± 11.18 (20)	31.83 ± 7.55 (6)	33.00 ±9.75 (8)	33.11 ± 11.76 (9)	36.72 ± 10.36 (18)	35.31 ± 8.18 (13)
t-test		*t*(65)=-.451 *p*=.654	*t*(30)=-.808 *p*=.425	*t*(28)=.540, *p*=.594	*t*(14)=-.082, *p*=.935	*t*(16)=-.020, *p*=.984	*t*(17)=-.322, *p*=.751	*t*(26)=-1.24, *p*=.227	*t*(21)=-.659, *p*=.348
STAI-X1 (n)	53.90 ± 14.10 (10)	55.47 ± 11.38 (57)	55.73± 14.36 (22)	51.80 ± 10.19 (20)	53.50 ± 14.54 (6)	55.50 ± 17.07 (8)	55.56 ± 9.07 (9)	56,94 ± 13.63 (18)	54.46 ± 13.94 (13)
t-test		*t*(65)= -.389 *p*=.698	*t*(30)=-.335 *p*=.740	*t*(28)=.468, *p*=.643	*t*(14)=.054, *p*=.957	*t*(16)=-.271, *p*=.790	*t*(17)=-.300, *p*=.768	*t*(26)=-5.60, *p*=.581	*t*(21)=-.095, *p*=.925
STAI-X2 (n)	57.80 ± 13.06 (10)	60.93 ± 8.25 (57)	60.68 ±09.78 (22)	59.25 ± 7.52 (20)	54.83 ± 10.80 (6)	63.13 ± 11.09 (8)	63.11 ± 4.73 (9)	61.39 ± 10.262 (18)	13.06 ± 9.45 (13)
t-test		*t*(65)=-1.01 *p*=.318	*t*(30)=-.739 *p*=.466	*t*(28)=-.388, *p*=.701	*t*(14)=.467, *p*=.648	*t*(16)=-.785, *p*=.444	*t*(17)=-1.15, *p*=.266	*t*(26)=-.805, *p*=.428	*t*(21)=-.404, *p*=.690
Cortical LDAEP (n)	0.291 ± 0.160 (9)	0.242 ± 0.180 (53)	0.289 ± 0.215 (19)	0.289 ± 0.195 (17)	0.380 ± 0.299 (6)	0.290 ± 0.174 (7)	0.147 ±0.115 (7)	0.281 ± 0.211 (15)	0.237 ± 0.150 (11)
t-test		*t(*55)=.589 *p*=.559	*t*(26)=.040 *p*=.969	*t*(24)=.030, *p*=.976	*t*(13)=-.754, *p*=.464	*t*(14)=-.300, *p*=.768	*t*(14)=1.99, *p*=.067	*t*(22)=.128, *p*=.900	*t*(18)=.755, *p*=.448
Source LDAEP (left) (n)	0.280± 0.173 (9)	0.204 ± 0.220 (53)	0.229 ± 0.283 (19)	0.216 ±0.212 (17)	0.398 ±0.461 (6)	0.141 ± 0.099 (7)	0.257 ±0.343 (7)	0.248 ± 0.198 (15)	0.114 ± 0.135 (11)
t-test		*t*(55)=.852 *p*=.398	*t*(26)= .496 *p*=.624	*t*(24)=.779, *p*=.444	*t*(13)=-.710, *p*=.490	*t*(14)=1.07, *p*=-.303	*t*(14)=.175, *p*=.864	*t*(22)=.460, *p*=.650	*t*(15)=2.36, ***p*=.032***
Source LDAEP (right) (n)	0.176 ±0.080 (9)	0.200 ± 0.247 (53)	0.148± 0.230 (19)	0.141 ±0.146 (17)	0.248 ± 0.381 (6)	0.074 ± 0.073 (7)	0.124 ± 0.093 (7)	0.205 ±0.159 (15)	0.211 ± 0.342 (11)
t-test		*t*(55)=-.367 *p*=.715	*t*(26)=.388 *p*=.701	*t*(24)=.094, *p*=.924	*t*(13)=-.565, *p*=.582	*t*(14)=1.88, *p*=.081	*t*(14)=1.19, *p*=.255	*t*(22)=-.510, *p*=.615	*t*(18)=-.302, *p*=.766

*other antidepressants (AD) = tetracyclic and tricyclic antidepressants. * $p < .05$, ** $p < .01$, *** $p < .001$.
